# Burden of and Trends in urticaria from 1990 to 2021 in China: A time-trend analysis and comparison with the United States and United Kingdom based on the GBD 2021

**DOI:** 10.1371/journal.pone.0330866

**Published:** 2025-08-29

**Authors:** Wenxi Xiao, Lizhi Xiang, Caizhi Peng

**Affiliations:** 1 Department of Dermatology, Wuhan Third Hospital, Wuhan, People’s Republic of China; 2 Department of Nursing, Wuhan Tongji Aerospace City Hospital, Wuhan, Hubei, China; Mashhad University of Medical Sciences, IRAN, ISLAMIC REPUBLIC OF

## Abstract

**Background:**

As the world’s largest developing country, China is facing a huge burden of urticaria. Studying the variations in urticaria management strategies between China and developed nations could offer valuable insights for policy development and enhance urticaria control efforts.

**Methods:**

We used the Global Burden of Diseases, Injuries, and Risk Factors Study (GBD) to acquire a detailed comprehension of the rates and trends of urticaria incidence and prevalence, DALYs burden, and control strategies in China and compared them with those in the United States and the United Kingdom.

**Results:**

In global both sexes, from 1990 to 2021, the all ages incidence of urticaria, age-standardized percent prevalence, and all ages DALYs cases increased from 84.8 (106, 95% UI: 74.5–96.2) to 117.0 (106, 95% UI: 104.1–131.0), 0.89% (95% UI: 0.79%−1.00%) to 0.90% (95% UI: 0.80%−1.02%), and 2.9 (106, 95% UI: 1.9–4.1) to 4.0 (106, 95% UI: 2.6–5.7), respectively. Meanwhile, the age-standardized incidence rate increased from 1529.2 (95% UI: 1355.9–1720.36) in 1990 to 1533.71 (95% UI:1358.4–1726.1) in 2021. The incidence counts and prevalence peaked in individuals aged 5–9 years for both males and females, and it shows a gradually decreasing trend in subsequent age groups. The number of incidences has shown a gradually increasing trend and the incidence was higher in female than male. In China, from 1990 to 2021, the all ages incidence of urticaria, age-standardized percent prevalence, and all ages DALYs cases increased from 16.06 (10^6^, 95% UI: 13.97–18.23) to 17.30 (10^6^, 95% UI: 15.37–19.25), 0.81% (95% UI: 0.71%−0.92%) to 0.79% (95% UI: 0.69%−0.90%), and 551.3 (10^3^, 95% UI: 362.6–792.7) to 591.8 (10^3^, 95% UI: 391.4–830.1), respectively. In general, over the past 30 years, urticaria has shown a slow growth trend in China. In the same period, age-standardized incidence rate (ASIR) decreased from 1342.4 (95% UI: 1176.0–1520.5) in 1990 to 1337.6 (95% UI: 1172.3–1514.7) in 2021.

**Conclusion:**

Clinical data for special populations such as the elderly, children, and pregnant women need to be further expanded and strengthened in China. High quality randomized controlled trial research is still needed for keeping pace with advancements.

## Introduction

Urticaria is a condition in which wheals (hives), angioedema, or both develop [1]. Acute form affects 20% of the general population and chronic urticaria (CU) up to 5% [2]. Clinically, it presents as varying sizes of wind masses accompanied by itching, and about 20% of patients have vascular edema [2]. Chronic urticaria refers to the daily or intermittent onset of wheals, lasting for more than 6 weeks [3]. Along with the physical symptoms, urticaria can greatly impact a patient’s quality of life by affecting their social and psychological well-being. In the past few years, urticaria has increasingly attracted the attention of clinical doctors and researchers. Pruritus had the greatest negative effect on the quality of life of patients with acute urticaria. In individuals with chronic urticaria, levels of mental health, physical impairment, activity both at work and outside of work, and anxiety and depression were similar to those of patients with moderate to severe psoriasis [4]. It is essential to have a thorough understanding of the global epidemiology of urticaria in order to enhance prevention and treatment methods and minimize its effects on public health.

The risk factors for acute urticaria include high population density, low economic income, and low hygiene status, while CU is associated with high income and social status [5], which may contribute to the heterogeneity of urticaria incidence, prevalence, and disability-adjusted life years (DALYs) burden. In developed countries, EAACI/GA2LEN/EDF/WO and AAAAI/ACAAI guidelines help optimize the establishment of urticaria control systems. However, most developing countries have limited information about urticaria. As the world’s largest developing country, China is facing a huge burden of urticaria. Study reported that the lifetime prevalence of urticaria was 7.30% and the point prevalence was 0.75% in the Chinese population [6]. A recent meta-analysis found that Asian showed a higher point prevalence of chronic urticaria (1.4%) than those from Europe (0.2–1.0) and Northern American (0.1%) [7]. China has gone through changes in its social, economic, and environmental sectors [7]. Lifestyle factors, including dietary habits and physical activity, have seen significant transformations. To adapt to the current socio-economic situation in China, it is necessary to establish a sound and effective urticaria management system. This elevated burden may be partially attributed to China’s rapid urbanization and associated environmental shifts over recent decades. Accelerated urbanization has led to increased exposure to novel environmental allergens, air pollutants (e.g., PM2.5, NO2), and lifestyle changes, all implicated in the development and exacerbation of allergic diseases including urticaria. The management strategies implemented by the United States and the United Kingdom have important reference significance for China. Therefore, the comparison of urticaria incidence rate, prevalence and DALYs between China and these developed countries can provide useful information for urticaria management. Today, the Global Burden of Diseases, Injuries, and Risk Factors Study (GBD) provides an opportunity to acquire a detailed comprehension of the rates and trends of urticaria incidence and prevalence, DALYs burden, and control strategies in China and compared them with those in the United States and the United Kingdom. We aim to provide valuable insights for policy planning and enhance urticaria management in China by studying the variations in control strategies across different countries.

## Materials and methods

### GBD source

The incidence, prevalence and DALYs caused by urticaria in China, the United States, and the United Kingdom in 1990 and 2021 were retrieved from the GBD 2021 online results tool, developed by the Institute for Health Metrics and Evaluation (IHME), which assesses illness, injury, and risk factor burden in 204 nations and territories [8]. DALYs were calculated by summing years lived with disability and years of life lost were computed by adding Years of Live Lost (YLLs) and Years Lived with Disability (YLDs) for each cause. Rates were standardized to the GBD world population and reported as age-standardized DALY rates, age-standardized incidence rates per 100,000 people. Urticaria were defined as the International Classification of Diseases (ICD-10: L50). DisMod-MR 2.1, a Bayesian meta-regression tool created by the IHME for modeling disease epidemiology, was utilized to determine the prevalence, incidence, and DALYs of urticaria. This tool enables the incorporation of different data sources such as surveys, administrative records, and published studies, while also adjusting for potential biases and heterogeneity in the data. The current GBD estimation is based on the methodology described in the latest GBD study, with more information available elsewhere; a brief overview is given below [9]. The current study presented the estimates of DALYs per 100,000 population in 2021 for Urticaria in China, US, and UK broken down by sex and age. Changes in percentage and ranking of all-age and age-standardized incidence rates from 1990 to 2021 were displayed to demonstrate the evolving trends of cancer burden in China, United States, and the United Kingdom.

### Statistical analysis

The rates of urticaria incidence, prevalence, and DALYs were compared between populations after adjusting for potential age structure confounding. This was done by standardizing the rates for each location, gender, and year to a GBD world standard population. All statistical analyses were conducted using R software (R 4.1.2 software). Our research utilized data obtained from the 2021 GBD study through secondary analysis. This study compiled information from different primary sources such as surveys, censuses, vital statistics, and health-related data sets that had already been approved by their institutional review boards. Since our analysis is based on aggregated and anonymized secondary data, there was no need for us to seek separate approval or exemption from an institutional review board.

## Results

### Urticaria burden in global

**Fig 1:**
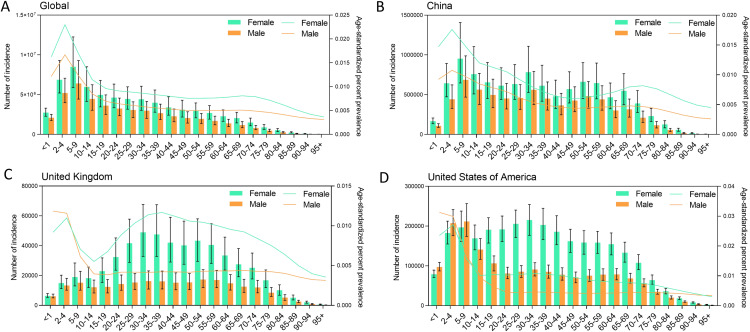
The all-ages number of incidences urticaria and age-standardized rate of incidence by age. **A.** Global; **B.** China; **C.** United Kingdom; **D.** United States.

**Fig 2:**
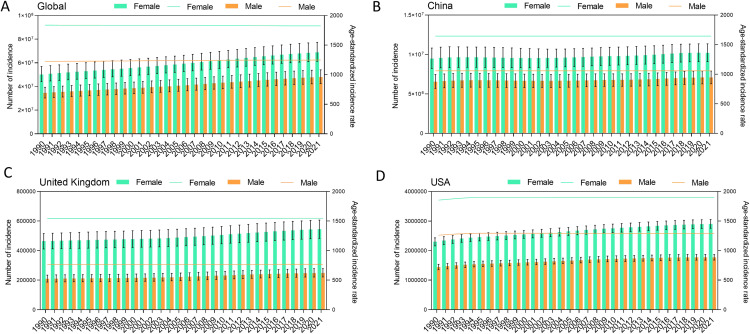
The all-ages number of incidences urticaria by year. **A.** Global; **B.** China; **C.** United Kingdom; **D.** United States.

In global both sexes, from 1990 to 2021, the all ages incidence of urticaria, age-standardized percent prevalence, and all ages DALYs cases increased from 84.8 (10^6^, 95% UI: 74.5–96.2) to 117.0 (10^6^, 95% UI: 104.1–131.0), 0.89% (95% UI: 0.79%−1.00%) to 0.90% (95% UI: 0.80%−1.02%), and 2.9 (10^6^, 95% UI: 1.9–4.1) to 4.0 (10^6^, 95% UI: 2.6–5.7), respectively. Meanwhile, the age-standardized incidence rate increased from 1529.2 (95% UI: 1355.9–1720.36) in 1990 to 1533.71 (95% UI:1358.4–1726.1) in 2021. The incidence counts and prevalence peaked in individuals aged 5–9 years for both males and females, and it shows a gradually decreasing trend in subsequent age groups **Fig 1A**. The number of incidences has shown a gradually increasing trend and the incidence was higher in female than male **Fig 2A**.

### Urticaria burden in China

In China, from 1990 to 2021, the all ages incidence of urticaria, age-standardized percent prevalence, and all ages DALYs cases increased from 16.06 (10^6^, 95% UI: 13.97–18.23) to 17.30 (10^6^, 95% UI: 15.37–19.25), 0.81% (95% UI: 0.71%−0.92%) to 0.79% (95% UI: 0.69%−0.90%), and 551.3 (10^3^, 95% UI: 362.6–792.7) to 591.8 (10^3^, 95% UI: 391.4–830.1), respectively. In general, over the past 30 years, urticaria has shown a slow growth trend in China **Fig 2B**. In the same period, age-standardized incidence rate (ASIR) decreased from 1342.4 (95% UI: 1176.0–1520.5) in 1990 to 1337.6 (95% UI: 1172.3–1514.7) in 2021. There was some variation in age-related sex-specific patterns. The incidence, prevalence, and DALYs was higher in female than male in 2021. In female, the incidence, prevalence, and DALYs was 10.22 (10^6^, 95% UI: 9.09–11.36), 0.98% (95% UI:0.86–1.11), and 349.0 (10^3^, 95% UI: 222.9–487.4), respectively. In male, the incidence, prevalence, and DALYs was 7.09 (10^6^, 95% UI: 6.28–7.91), 0.66% (95% UI:0.57–0.74), and 243.6 (10^3^, 95% UI: 160.2–338.9), respectively. The incidence counts peaked in individuals aged 5–9 years for both males and females. Notably, the incidence of children and adolescents and adults is equivalent. Moreover, there is a noticeable downward trend starting from the age of 50 **Fig 1B and Table 1**. Surprisingly, the number of incidences has remained largely unchanged in the past 30 years in China.

**Table 1:** The all-ages incidence of urticaria, age-standardized percent prevalence, and all ages DALYs cases of 2021 in China.

Items	Male			Female		
	Incidence, N (95% UI)	Prevalence, rate (95% UI)	DALYs, N (95% UI)	Incidence, N (95% UI)	Prevalence, % (95% UI)	DALYs, N (95% UI)
**Age groups**						
<1	108519.22(88190.95,135385.22)	523.14(427.99,638.78)	2013.37(1261.89,2949.87)	167189.57(136083.68,206932.94)	942.02(766.73,1166.44)	3116.57(1957.8,4551.57)
2–4	443377.96(325298.96,623167.3)	908.35(694.7,1186.92)	16000.19(9769.09,24738.31)	644257.35(473146.41,893623.43)	1545.55(1180.7,2021.62)	23643.57(14475.37,35543.47)
5–9	686467.28(469748.42,988483.21)	785.24(544.84,1154.6)	24778.1(14114.1,39485.26)	952545.06(646467.1,1406864.6)	1248.72(869.47,1808.62)	34686.71(19964.84,54024.99)
10–14	563944.81(376373.27,767535.57)	702.04(471.43,990.34)	19913.89(11025.36,31626.09)	758991.47(509666.11,1104033.12)	1080.93(736.32,1550.32)	26737.09(15194.78,42336.07)
15–19	496447.97(336323.26,695324.79)	707.59(490.43,980.56)	17464.4(10135.16,28930.52)	656038.35(451758.75,904552.78)	1084.58(758.65,1513.73)	23052.23(13623.99,37892.28)
20–24	452706.41(320816,641320.94)	675.13(470.16,941.09)	16141.84(9156.77,26254.74)	613777.91(440925.75,838274.74)	1035.01(720.33,1418.04)	21647.78(12545.3,35204.86)
25–29	459514.96(315232.05,626304.85)	586.65(402.81,813.35)	16427.83(9179.68,26586.47)	634576.99(440864.51,878056.76)	906.68(629.12,1280.28)	22558.1(12319.94,36963.68)
30–34	561428.96(375290.43,791972.88)	517.93(348.46,739.86)	19737.42(11670.29,32173.78)	784381.61(528583.19,1109835.77)	780.33(515.62,1104.84)	27485.79(16523.77,43214.61)
35–39	451751.25(306277.92,660420.29)	477.56(327.44,685.92)	15748.02(9107.16,25284.79)	613496.46(418518.21,870197.34)	688.18(476.68,992.26)	21295.61(12458.67,33320.34)
40–44	365480.3(246313.07,516882.2)	448.49(294.46,655.26)	12690.95(7006.17,21679.39)	480231.85(326323.46,683084.48)	621.86(410.5,904.46)	16514.39(9043.4,27438.82)
45–49	427327.03(287249.94,598958.84)	434.63(292.4,611.25)	14582.73(8471.02,23336.85)	570561.17(395925.09,787584.36)	598.7(411.22,822.04)	19234.33(10917.56,29744.86)
50–54	483384.12(330056.03,664409.87)	448.17(295.33,620.86)	16232.89(9190.09,25754.68)	663414.96(450295.67,902882.45)	627.99(415.19,858.77)	21986.87(12578.98,34511.05)
55–59	441425.51(305687.6,611927.23)	458.22(316.78,633.7)	14748.3(8835.95,22360.8)	646491.16(447922.93,897827.76)	664.09(455.82,913.89)	21240.49(12732.53,32654.2)
60–64	301942.67(210421.19,424456.67)	465.13(324.67,661.4)	9888.83(5803.63,15526.68)	468310.24(322751.7,646944.42)	719.47(503.22,1007.92)	15008.72(8730.85,23042.08)
65–69	317388.45(218259.48,445753.43)	479.72(321.81,684.64)	10323.41(5966.46,16131.16)	549826.21(374350.64,764479.15)	797.89(532.59,1130.32)	17556.4(10106.31,26815.81)
70–74	210829.46(150445.34,294651.95)	468.37(328.34,651.34)	6776.22(4069.49,10485.06)	391181.83(276369.56,552444.11)	817.2(574.48,1151.85)	12421.69(7417.93,19267.33)
75–79	116877.22(81768.21,162584.2)	434.86(297.62,611.93)	3724.02(2216.88,5909.52)	231297.23(162531.3,326526.77)	766.45(524.87,1100.48)	7269.96(4324.36,11269.9)
80–84	56400.55(36756.64,77308.38)	379.03(245.01,525.41)	1766.69(1052.05,2764.55)	127168.92(83695.16,173130.54)	667.14(433.01,917.04)	3919.91(2310.83,5926.78)
85–89	19240.98(13547.32,25881.22)	323.01(219.65,442.36)	593.53(344.22,906.72)	58421.82(40652,78183.9)	565.29(383.53,768.59)	1764.94(1083.19,2722.54)
90–94	3939.14(2767.04,5339.48)	281.31(191.39,385.43)	118.9(69.58,184.65)	17995.96(12525.37,24417.17)	489.76(338.2,673.74)	525.34(300.87,807.81)
95+	545.66(353.04,812.77)	260.06(176.28,373.39)	16.14(9.54,25.85)	4049.01(2611.19,5996.22)	450.47(303.47,639.51)	113.84(68.34,179.72)

### Urticaria burden in United Kingdom and United States

In United Kingdom, from 1990 to 2021, the all ages incidence of urticaria, age-standardized percent prevalence, and all ages DALYs cases increased from 0.21 (10^6^, 95% UI: 0.19–0.23) to 0.25 (10^6^, 95% UI: 0.22–0.28), 0.70% (95% UI: 0.62%−0.79%) to 0.71% (95% UI: 0.63%−0.80%), and 22.5 (10^3^, 95% UI: 14.9–31.0) to 26.5 (10^3^, 95% UI: 17.7–36.3), respectively. The number of cases shows an upward trend before the age of 35–39, followed by a downward trend, and from around 15 years old, the number of cases in female is about twice that of male **Fig 1C**. In United States of America, from 1990 to 20121, the all ages incidence of urticaria, age-standardized percent prevalence, and all ages DALYs cases increased from 3.7 (10^6^, 95% UI: 3.5–4.0) to 4.7 (10^6^, 95% UI: 4.4–4.9), 0.96% (95% UI: 0.90%−1.02%) to 0.98% (95% UI: 0.93%−1.03%), and 126.9 (10^3^, 95% UI: 85.3–175.1) to 156.7 (10^3^, 95% UI: 106.7–216.6), respectively. Interestingly, the incidence counts peaked in individuals aged 1–4 years for both males and females. Before the age of 9, the number of cases in males is greater than that in females **Fig 1D**. Over the past 30 years, urticaria has shown a slow growth trend both in United Kingdom and United States.

## Discussion

This study involved a thorough epidemiological analysis of the incidence, prevalence, and DALYs impact of urticaria in China, utilizing data from the GBD 2021. Furthermore, we examined the changes in urticaria incidence over the last thirty years and compared the disparities among China, United Kingdom, and United States by utilizing reliable data from the GBD 2021 database. We found that China has higher urticaria incidence and DALYs than the United Kingdom and United States in 2021. While United States has higher urticaria prevalence than China and United Kingdom in 2021. Female had higher urticaria rates across all age categories except the 1–9 age group in United States, which the number of cases in males is greater than that in females. From 1990 to 2021, there was an increase in disease burden, prevalence, and incidence in most age groups, with a focus on younger individuals and female. The observed increase in China’s all-ages incidence count of urticaria occurred alongside stable ASIR. This divergence primarily reflects demographic dynamics: 1) Population Growth: China’s significant population expansion during this period naturally increased the absolute number of cases, even if individual disease risk remained constant; 2) Aging Population Structure: China underwent substantial demographic aging. As urticaria incidence peaks in children/young adults (5–9 years) and declines with age, the proportional decrease in younger, higher-risk age groups within the overall population counterbalanced the effect of population growth on the rate. Consequently, the ASIR, which adjusts for differences in age distribution over time and between populations remained stable, indicating that the per capita risk of developing urticaria for a standardized population did not significantly change at the national level over these three decades. The rising absolute burden is thus largely attributable to population growth interacting with shifting demographics. The high incidence rate and high DALYs of urticaria in China mainly reflect the increasing disability caused by a large population, increased environmental incentives and insufficient treatment; The relatively low incidence rate highlights the weak management of chronic cases and differences in medical accessibility, resulting in patients not being consistently included in the statistics. This contradictory phenomenon is essentially a common manifestation of the differences in the medical system and the dynamic characteristics of diseases, which suggests that China needs to strengthen chronic disease management and research on special populations to reduce cumulative burden.

Our study found that female have a higher burden of urticaria compared to male, which aligns with previous research indicating a higher prevalence of autoimmune diseases in female [10]. Several factors, such as hormonal differences, genetic predispositions, and environmental exposures, may contribute to this disparity. Estrogen levels, known to regulate immune responses, could be a factor in women’s increased susceptibility to autoimmune diseases [11]. Additionally, genetic factors on the X chromosome may also play a role in the higher risk of autoimmune diseases in female [12]. The implementation of population-based urticaria screening programs is one of the most effective measures adopted by the United Kingdom and United States, especially in treatment approach. For example, in United States, the treatment of urticaria in pregnant or lactating women and children is mostly similar to that of nonpregnant adults. Antihistamines should be used in pregnant or lactating women at the lowest effective dose for the shortest duration possible. In children, it is preferable to use second-generation antihistamines over first-generation ones and to avoid or use corticosteroids sparingly [13]. In the United Kingdom, urticaria and angio‐oedema show greater similarities in children and adolescents than in adults, with similar treatment approaches. There is not enough evidence to confidently recommend the safety of most medications during pregnancy and while breastfeeding [14]. It is worth noting that, the incidence counts peaked in United States children, while the incidence counts peaked in United Kingdom adult. The age onset pattern in China is similar to that in the United Kingdom, but the situation of treatment control in our country is still not ideal. Due to insufficient clinical research targeting the Chinese population, the effectiveness and safety of increasing doses of first generation and second-generation antihistamines in specific populations have not been well evaluated. Therefore, clinical data for special populations such as the elderly, children, and pregnant women need to be further expanded and strengthened in China.

The main cause of adult CU is more likely to be due to autoimmune factors, among which anti IgE monoclonal antibodies (omazumab) have shown broad clinical prospects. Omazumab was approved in multiple countries in the United States and Europe in 2014 for use in adults and adolescents (≥12 years old) with chronic spontaneous urticaria (CSU) who are resistant to antihistamines [15]. In 2017, omazumab was approved for the treatment of adult and adolescent (≥12 years old) patients with moderate to severe allergic asthma and entered China [16]. In china, researches demonstrated the efficacy and safety of omalizumab in Chinese adult patients with chronic spontaneous urticaria who remained symptomatic despite H1AH therapy [17]. Although not licensed in the United Kingdom, omalizumab has been successfully used in children below the age of 12 years with chronic spontaneous urticaria and inducible urticarias, the 2018 Chinese Urticaria Guidelines also recommend Omizumab as one of the options for third line treatment for patients with antihistamine resistance. In China, high quality randomized controlled trial research is still lacking, and the treatment of CU in China is still beyond the prescribed medication. China’s implementation of new drugs and therapeutic strategies is not keeping pace with advancements. The experience of the UK and the US provides a key path for China to develop localized guidelines for urticaria in special populations: priority should be given to establishing electronic case registration systems for the elderly, children, and pregnant women to track real-world efficacy, and developing age stratified medication standards for children based on the UK and US models, combined with the Chinese medical insurance system to optimize the admission of biologics.

China is undergoing a transformation of unhealthy lifestyles, there has been a move towards diets consisting of more animal-based foods, refined grains, and highly processed foods, as well as a shift towards more sedentary lifestyles [18]. The European guidelines, United Kingdom and United States do not recommend dietary control urticaria, as several patients seek dietary modifications as they are easy and cost-effective. The Chinese guidelines recommend that patients with suspected food related urticaria should be encouraged to keep a food diary, search for possible food allergens, and avoid them. CSU patients often consider adjusting their diet and discussing potential food triggers. However, the pathogenic role of food in CSU is still unclear. Chinese people like to eat high-fat red meat (pigs, cows, sheep), while the diet of the United Kingdom and United States is paired with a large number of fresh vegetables and fruits every day, with chicken and beef as the main meat. A personalized diet is created by reviewing a person’s personal history and food journal, focusing on eliminating potential triggers. It is important to tailor the diet to include specific foods that the individual is allergic to, such as avoiding all nuts for those allergic to peanuts, rather than implementing a broad restriction on all nuts. The elevated incidence of childhood allergies observed in the United States likely stems from a combination of altered allergen exposure patterns and heightened diagnostic sensitivity, interacting with environmental and immunological factors. Shifts in dietary habits, hygiene practices, and environmental exposures (e.g., pollution, reduced microbial diversity) may contribute to increased sensitization risk and altered immune development in children. Concurrently, widespread access to and utilization of sensitive diagnostic tests (like skin prick tests and specific IgE blood tests) in the U.S. healthcare system detects subclinical sensitizations that might not manifest as clinical disease, potentially inflating reported prevalence estimates. The present study has several limitations. First, the comparison of estimates from different countries may be compromised because of variations in data collection and reporting systems across countries. Second, it is difficult to estimate the reasons for the differences among different age groups in China, the United Kingdom, and the United States, especially, the number of cases in males is greater than that in females before the age of 9 in United States.

## Conclusion

China has higher urticaria incidence and DALYs than the United Kingdom and United States in 2021. While United States has higher urticaria prevalence than China and United Kingdom in 2021. From 1990 to 2021, there was an increase in disease burden, prevalence, and incidence in most age groups, with a focus on younger individuals and female. Clinical data for special populations such as the elderly, children, and pregnant women need to be further expanded and strengthened in China. High quality randomized controlled trial research is still needed for keeping pace with advancements.
